# Unraveling Genetic Diversity Amongst European Hazelnut (*Corylus avellana* L.) Varieties in Turkey

**DOI:** 10.3389/fpls.2021.661274

**Published:** 2021-07-01

**Authors:** Nihal Oztolan-Erol, Andrew J. Helmstetter, Asuman İnan, Richard J. A. Buggs, Stuart J. Lucas

**Affiliations:** ^1^Sabancı University Nanotechnology Research and Application Center, İstanbul, Turkey; ^2^Jodrell Laboratory, Royal Botanic Gardens, Kew, United Kingdom; ^3^School of Biological and Chemical Sciences, Queen Mary University of London, London, United Kingdom

**Keywords:** hazelnut (*Corylus avellana* L.), private alleles, genetic diversity, RAD-seq, SNP identification

## Abstract

European hazelnut (*Corylus avellana*) is a diploid (2n = 22), monecious and wind-pollinated species, extensively cultivated for its nuts. Turkey is the world-leading producer of hazelnut, supplying 70–80% of the world’s export capacity. Hazelnut is mostly grown in the Black Sea Region, and maintained largely through clonal propagation. Understanding the genetic variation between hazelnut varieties, and defining variety-specific and disease resistance-associated alleles, would facilitate hazelnut breeding in Turkey. Widely grown varieties ‘Karafındık’ (2), ‘Sarıfındık’ (5), and ‘Yomra’ (2) were collected from Akçakoca in the west, while ‘Tombul’ (8), ‘Çakıldak’ (3), ‘Mincane’ (2), ‘Allahverdi’ (2), ‘Sivri’ (4), and ‘Palaz’ (5) were collected from Ordu and Giresun provinces in the east (numbers in parentheses indicate sample sizes for each variety). Powdery mildew resistant and susceptible hazelnut genotypes were collected from the field gene bank and heavily infected orchards in Giresun. Every individual was subjected to double digest restriction enzyme-associated DNA sequencing (ddRAD-seq) and a RADtag library was created. RADtags were aligned to the ‘Tombul’ reference genome, and Stacks software used to identify polymorphisms. 101 private and six common alleles from nine hazelnut varieties, four private from resistants and only one from susceptible were identified for diagnosis of either a certain hazelnut variety or powdery mildew resistance. Phylogenetic analysis and population structure calculations indicated that ‘Mincane’, ‘Sarıfındık’, ‘Tombul’, ‘Çakıldak’, and ‘Palaz’ were genetically close to each other; however, individuals within every varietal group were found in different sub-populations. Our findings indicated that years of clonal propagation of some preferred varieties across the Black Sea Region has resulted in admixed sub-populations and great genetic diversity within each variety. This impedes the development of a true breeding variety. For example, ‘Tombul’ is the most favored Turkish variety because of its high quality nuts, but an elite ‘Tombul’ line does not yet exist. This situation continues due to the lack of a breed protection program for commercially valuable hazelnut varieties. This study provides molecular markers suitable for establishing such a program.

## Introduction

European hazelnut (*Corylus avellana* L.) is a diploid (2n = 22), monecious, dichogamous, self-incompatible, perennial, wind-pollinated species belonging to the Betulaceae family, and can be grown in bush form or from a single trunk (Brown et al., 2016; Öztürk et al., 2017b). European hazelnut is commercially important for its nuts. It is cultivated in northern Europe from southern Norway and Finland in the west, to the Ural Mountains to the east, and further south from western Iberia and Morocco to the Black Sea region of Turkey (Brown et al., 2016). Turkey is the world-leading producer of hazelnuts with 80% of cultivated area in the world (Sezer et al., 2017). Hazelnut is grown throughout the Black Sea and the Eastern Marmara regions of Turkey, where 90% of hazelnut cultivation is maintained in the provinces of Ordu, Trabzon, Giresun, Samsun, Duzce, and Sakarya (Sezer et al., 2017).

Despite its significant economic return, the official reports note that Turkey lags behind its competitors (Italy, Azerbaijan, Iran, and Georgia) in terms of hazelnut production per unit area (Okay et al., 1985; Erdoğan, 2018; TMO, 2019). Traditional propagation practices, changing climate conditions and prevalent diseases are reported as reasons for decreasing hazelnut production. The traditional hazelnut propagation practice in Turkey for centuries has been clonal multiplication of healthy and productive trees through suckers (Erdoğan, 2018; İslam, 2018). In Turkish orchards, hazelnut is usually found as multi-stemmed bushes. A circular sucker planting system, which is called an “Ocak”, is the traditional hazelnut planting method in Turkey. Spacing between Ocaks and between stems within the Ocak strongly affects the yield capacity of orchards (Beyhan et al., 2020). Dense planting of stems in an Ocak restricts necessary practices such as pruning, harvesting, and applying disease treatments, and reduces yield. A sparse planting in Ocaks including up to five or six stems and 4 m spacing between every Ocak is recommended by the Ministry of Agriculture and Forestry Hazelnut Research Institute (Okay et al., 1985). As an alternative, “hedge planting” with lines of single trunks at 1.5–2 m intervals has been proposed by the institute; this promotes higher yield, easier pruning, harvesting, fertilizing and application of disease treatments. Another advantage of the hedge planting system on steep terrain is that it requires a narrower terrace spacing than the Ocak system, reducing labor costs (Okay et al., 1985). Orchard renewal and remodeling has high potential to improve hazelnut yields in Turkey, creating a need for well-characterized and locally adapted varieties.

Climate factors and soil types greatly affect the growth of hazelnut trees. Mild summer and winter temperatures (below 36°C and above −8°C, respectively), adequate rainfall or irrigation are preferred climate conditions for productive hazelnut growth, along with deep, well-drained soil containing high organic matter (pH 6) (İslam, 2018). The average annual rainfall in the Black Sea Region is around 800–1000 mm and temperature changes in average between 8 and 21°C. Many orchards in the eastern Black Sea region have shallow soils, therefore yield/hectare is actually very low. On the other hand, climate conditions provide a suitable habitat for hazelnut trees, even though late spring frost and fungus infection might negatively affect the hazelnut production in this area (Sezer et al., 2017; Lucas et al., 2018, 2020).

Powdery mildew is a widespread disease of hazelnut in the Black Sea region. Two different powdery mildew causing agents have been identified (Sezer et al., 2017). *Phyllactinia guttata*, a member of Erysiphaceae family, is a widespread infectious fungus worldwide with mild symptoms, which does not affect hazelnut production. On the other hand, another fungus, *Erysiphe corylacearum*, was first reported in the eastern Black Sea region in 2013 and has now spread throughout the hazelnut cultivation areas, showing severe symptoms and reduction in hazelnut yields (Lucas et al., 2018). As another member of the Erysiphaceae family, *E. corylacearum* shows distinct effects from *P. guttata* and has previously been identified on various *Corylus* species in the Asia and the North America. Natural variation among hazelnut cultivars and wild individuals can provide hazelnut trees resistant to powdery mildew disease. Identification of alleles responsible for the resistant phenotype would help to prevent infection of the disease through a breeding program.

Rowley et al. (2012) published the first assembled and characterized European hazelnut draft genome for the cultivar ‘Jefferson’ (Mehlenbacher et al., 2011; Sathuvalli and Mehlenbacher, 2011; Rowley et al., 2012). *C. avellana* cv. ‘Jefferson’ was developed and released in 2009 by Oregon State University, and was selected for resistance to Eastern Filbert Blight (EFB) disease (Sathuvalli et al., 2017). Rowley et al. (2012) conducted *de novo* genome and transcriptome assemblies and achieved a 91% genome coverage for Jefferson (Rowley et al., 2012), which is currently being improved by incorporation of long-read sequencing technologies (Snelling et al., 2018). Recently, Revord et al. (2020) identified a core set of *Corylus americana*, a genetically and phenotypically diverse, cold hardy and EFB resistant hazelnut species using single nucleotide polymorphism (SNP) markers. This research provided diverse genetic resources for improving hazelnut production, as *C. americana* is cross-compatible with *C. avellana* (Revord et al., 2020). Meanwhile, Lucas et al. (2020) have recently published a chromosome-scale genome assembly for Turkish *C. avellana* variety ‘Tombul’ giving 97.8% coverage of the estimated genome size with 370 Mb length and 11 pseudomolecules (Lucas et al., 2020). ‘Tombul’ is the most important hazelnut variety in Turkey due to its high nut quality in terms of taste and oil content, and high productivity. The ‘Tombul’ genome assembly was provided as a reference genome particularly relevant for molecular breeding projects to improve hazelnut production in Turkey.

Understanding the molecular genetic diversity of hazelnut in Turkey is essential to reaching this goal. Genetic diversity between cultivars grown in Black Sea countries (Turkey, Georgia, and Azerbaijan) was investigated previously using simple sequence repeat (SSR) markers (Gürcan et al., 2010). Results showed both that some varieties were similarly named although they were phenotypically and/or genotypically different, or differently named although they were clonally propagated. Randomly amplified polymorphic DNA (RAPD), intersimple sequence repeat (ISSR), and amplified fragment length polymorphism (AFLP) markers have also been used to characterize the relatedness between Turkish hazelnut varieties (Kafkas et al., 2009; Erdoğan et al., 2010). Öztürk et al. (2017b) also studied the Turkish national hazelnut collection to identify genetic diversity and population structure, as well as selecting a core set which includes the most diverse accessions. This collection consists of 402 different accessions collected from the Black Sea Region including cultivars, landraces and wild accessions, which were classified using SSR markers (Öztürk et al., 2017b). European and American hazelnut cultivars and/or wild accessions have also been assessed using a variety of molecular markers such as SSRs and AFLPs (Boccacci and Botta, 2009; Leinemann et al., 2013; Martins et al., 2013; Brown et al., 2016; Bhattarai and Mehlenbacher, 2018). However, these molecular markers are based on oligonucleotide probes hybridizing to specific loci on the DNA, and they might produce conflicting results in some varieties due to unexpected polymorphisms within the oligo binding sites (Wang et al., 2013).

As a cheaper and higher resolution technique, the combination of restriction sites with next generation sequencing (NGS) has eased the way to discover genome-wide polymorphisms for any species (Davey et al., 2011). Restriction-site-associated DNA sequencing (RAD-seq) is a very effective method to identify SNPs in a population lacking a well-assembled reference genome, which has been applied successfully in hazelnut (Torello Marinoni et al., 2018).

In our previous study, we used double digest restriction enzyme-associated DNA sequencing (ddRAD-seq) to investigate the genetic diversity and domestication of cultivated and wild hazelnuts in Turkey (Helmstetter et al., 2020). This included 200 individuals from cultivated and wild hazelnut trees collected in the Black Sea region in Turkey, along with related *Corylus* species and specimens from the United Kingdom, Georgia, and Italy. Population genetic analyses revealed that cultivated hazelnuts showed elevated heterozygosity compared to wild individuals, and that genetic similarity did not correlate well with cultivar names. This might be due to somatic mutations, propagation of natural hybrid hazelnuts germinated from fallen seeds, and/or propagation of a group of clones that physiologically look alike but are actually genotypically different. This suggested that clonal propagation has promoted outbreeding and genetic admixture of Turkish hazelnut varieties across the growing region. Therefore, in this study we refer to these as “varieties”, meaning assemblies of individuals with different genetic backgrounds but convergent phenotypes, propagated vegetatively; as distinct from “cultivars” that are produced from a deliberate breeding effort through multiple seed generations, which are therefore more genetically homogeneous.

Here, we have re-analyzed the cultivated hazelnut varieties (32 individuals) and also powdery mildew resistant (8) and susceptible (13) wild accessions (21 individuals) from the previous study; firstly, by aligning the RAD-tags to the recently completed *C. avellana* var. ‘Tombul’ reference genome (Lucas et al., 2020), allowing the chromosomal distribution of loci to be assessed. Secondly, we focused on identifying polymorphic alleles that are specific and shared between each of nine Turkish varieties, along with two mixed populations containing individuals that were resistant and susceptible to powdery mildew disease, respectively. By identifying private alleles (i.e., an allelic variant only observed in one variety) that are diagnostic for each variety and also for powdery mildew resistant hazels, we aim to provide a basis for developing molecular markers that will be useful in orchard renewal and future breeding programs.

## Materials and Methods

### Plant Sample Collection

**FIGURE 1:**
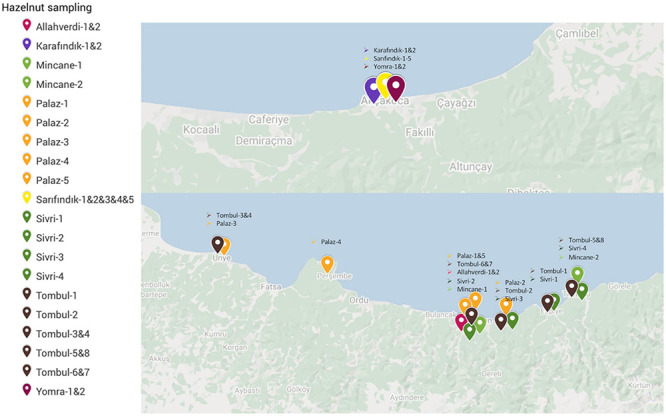
Locations from which samples of nine hazelnut varieties were collected in 2016–2017.

Cultivated hazelnut individuals were collected to analyze their genetic diversity. Fresh leaf buds were collected from both Eastern (Ordu, Giresun) and Western (Akçakoca) Black Sea provinces. ‘Karafındık’, ‘Sarıfındık’, and ‘Yomra’ (also named as Foşa in the Eastern Black Sea region) were collected from Akçakoca orchards, while ‘Allahverdi’, ‘Çakıldak’, ‘Mincane’, ‘Palaz’, ‘Sivri’, and ‘Tombul’ were collected from a wider geographic area in the Eastern Black Sea region (Figure 1). ‘Mincane’ and ‘Sarıfındık’ are reportedly closely related but named differently in the Eastern and the Western Black Sea regions (H. Irfan Balık, personal communication).

**FIGURE 2:**
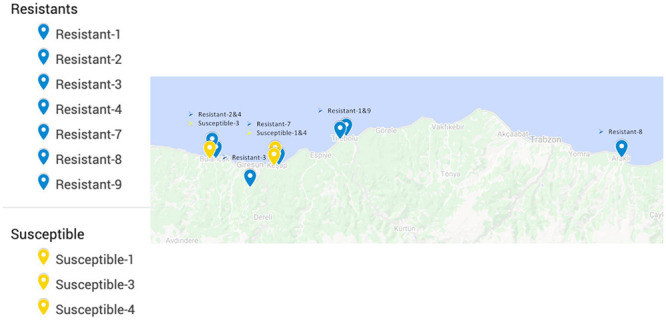
Locations from which resistant and susceptible hazelnut accessions originated, prior to transplantation to the field gene bank at Giresun Hazelnut Research Institute. Records of the original location for one resistant and several susceptible individuals were not available, so these are not shown on the map.

All of the commercial hazelnut varieties in Turkey are susceptible to powdery mildew infection (Lucas et al., 2018). Therefore, resistant accessions were selected from the non-cultivated landraces/wild individuals conserved in the field gene bank of the Giresun Hazelnut Research Institute. These accessions were largely collected between 1969 and 1972 from locations around the eastern Black Sea region (Figure 2), based on morphological diversity, and have been maintained by clonal propagation until the present day (Öztürk et al., 2017b). Mildew resistance phenotype was determined by scoring the prevalence of mildew on leaves as described previously (Lucas et al., 2018) over two growing seasons during which the majority of the gene bank was heavily infected; only eight accessions were found to have no signs of mildew and were selected as the “resistant” group. Accessions with similar geographic origins to the resistant individuals, but scored with the highest prevalence of mildew, were selected to form a “susceptible” group.

### DNA Extraction

DNA of each individual was extracted for RAD-seq analysis. A modified version of the DNA extraction protocol from Wang et al. (2013) was conducted using fresh leaf buds. 200–250 mg of tissue was added with one 5 mm bead into a 2 ml TissueLyser tube. Tubes were frozen at −80°C for at least 20 min and then beaten for 1 min at 30 s^–1^ until the buds were fully pulverized, and then DNA extraction carried out as described previously (Lucas et al., 2020). The isolated DNA was purified further by cleaning on spin columns from the Qiagen DNeasy Plant Miniprep kit.

### RAD-Seq Library Preparation

Barcoded ddRAD-seq libraries were constructed using *Eco*RI and *Msp*I in the Jodrell Laboratory (Royal Botanical Gardens, Kew, United Kingdom) as described previously. Ten PCR reactions were run for every library and then combined to minimize PCR bias, and batches of samples were normalized and then pooled in equivalent quantities before sequencing. Sequencing of 150 bp paired-end reads was realized on an Illumina HiSeq 4000 at the Edinburgh Genomics sequencing facility (Helmstetter et al., 2020). In order to demultiplex and clean the ddRAD-seq data, the *process_radtags* command was used using the cut sites of *Msp*I and *Eco*RI (Peterson et al., 2012). All demultiplexed ddRAD-seq data were uploaded to the European Nucleotide Archive (Project Accession no: PRJEB32239. Run accessions used in this study: ERR3293948; ERR3299084-3299095; ERR3299102-3299130; ERR3362869-3362889).

### RAD-Tag Alignment to a Draft Reference Genome

The chromosome-scale genome assembly of the ‘Tombul’ variety (GCA_901000735.1) reported by Lucas et al. (2020) was indexed using *bwa index* (Burrows-Wheeler Aligner). Next, the demultiplexed and cleaned reads were aligned to the indexed reference genome using *bwa mem*. and Sequence Alignment/Map (SAM) files created (Li et al., 2009).

### Stacks Reference Genome Pipeline

The Stacks reference genome pipeline was followed to analyze population genetics independently in the Turkish hazelnut varieties (*n* = 32) and resistant and susceptible accessions to powdery mildew disease (*n* = 21) (Catchen et al., 2013; Paris et al., 2017). The pipeline is summarized below and in Supplementary Figure 1.

(1) *pstacks*: Aligned data were grouped into loci and polymorphic nucleotide sites were identified for each individual.
(2) *cstacks*: Loci were grouped together across each individual. A consensus catalog was created.
(3) *sstacks*: Loci of each individual were tested if they matched against the consensus catalog.
(4) *rxstacks*: Genotype and haplotype corrections were made based on the population-wide accumulated data.
(5) *populations*: The population genetic statistics were calculated based on the population map showing which individuals belong to which population. Individuals that belong to a variety were grouped within the same population.

### Population Structure

The population structure was inferred by calculating the similarity between each individual’s haplotype using the *fineRADStructure* package in order to estimate co-ancestry across the individuals (Malinsky et al., 2018). Parameters were specified as follows: −n 10 (maximum number of SNPs allowed in a haplotype locus); −m 75 (cut-off value% of missing data in individuals). The *RADpainter* command was used to calculate the closest relatives for each allele from each RAD locus, these were then clustered by *fineRADStructure* using a Markov Chain Monte Carlo (MCMC) algorithm.

### Phylogenetic Analysis and Tree

The phylogenetic analysis was conducted using 32 individuals and 10645 variant sites that were present in every variety via Randomized Accelerated Maximum Likelihood (RAxML) (Stamatakis, 2014). The program was run through the command *raxmlHPC* using GTRGAMMA as model (−m) with 100 cycles (−#). The phylogenetic tree was built via FigTree software using best tree output with bootstrapping results.

### Polymorphic Gene Annotation

Gene modeling was performed using Augustus (Stanke et al., 2008) for *de novo* gene prediction using the ‘Tombul’ genome assembly, with conditions optimized for *Arabidopsis thaliana*. The sequences of Stacks loci containing private or shared alleles were mapped against the ‘Tombul’ gene models using BLASTN. The predicted coding sequences of matches were uploaded in the online *Mercator* tool, which assigned Gene Ontology terms to each sequence on the basis of sequence similarity searches (Lohse et al., 2014).

## Results

### Genotyping and Population Genetics Statistics

The genetic diversity of Turkish hazelnut varieties, along with resistant and susceptible accessions, were investigated through a series of statistical analyses. RAD-seq results were interpreted using Stacks reference genome pipeline. *cstacks* generated a set of consensus loci by merging alleles sequenced in multiple samples together. In total, 472,140 loci were generated, and every locus was 150 bp in length, with an average read depth of 75 at each locus. 1,048,575 SNPs were identified in the consensus catalog (Table 1). These loci were evenly distributed among all 11 assembled pseudochromosomes, with a mean average density of 1253 loci/Mb.

**TABLE 1:** Distribution of consensus ddRAD-tags and single nucleotide polymorphisms (SNPs) on chromosomes in the ‘Tombul’ genome assembly.

**Chromosomes**	**SNPs**	**Loci**	**Average no. of ddRAD loci/Mb**
1	134502	61813	1213
2	126956	60694	1193
3	99283	49135	1235
4	103804	46402	1259
5	97803	45901	1252
6	85327	36265	1198
7	85690	40451	1338
8	68405	31939	1239
9	68374	30931	1329
10	61963	29783	1310
11	63297	27213	1293
NA	53171	–	

The population genetics statistics were analyzed separately for Turkish hazelnut cultivars and resistant and susceptible accessions using the *populations* program in Stacks; total/mean values across all polymorphic loci are given in Table 2. Among the cultivated varieties, ‘Karafındık’ had the highest number of private alleles. Chi-squared tests showed that overall deviations from the expected homozygosity and heterozygosity values were not statistically significant (Table 2). The average *P* values (major allele frequency) ranged from 0.778 to 0.890. The lowest nucleotide diversity estimate (π) belonged to ‘Allahverdi’ variety and the highest to ‘Sarıfındık’. All of the sub-populations had observed heterozygosity higher than expected heterozygosity and in-breeding coefficients (F_IS_) close to 0, consistent with out-breeding dominating their recent genetic history.

**TABLE 2:** Summary of population genetic statistics summarized across all loci for each subpopulation: Number of private (Pr) alleles, number of individuals (N), the mean frequency of the most frequent allele at each locus (P), the observed (Obs) and the expected (Exp) homozygosity (Hom) and heterozygosity (Het), the mean value of estimated nucleotide diversity (π), and the mean inbreeding coefficient (F_IS_) across all loci, χ^2^ test statistic and corresponding *p*-values of hazelnut varieties (a) and resistant-susceptible accessions (b).

		**Pr Alleles**	**N**	**P**	**Obs Het**	**Obs Hom**	**Exp Het**	**Exp Hom**	**π**	**F_IS_**	**χ^2^ test**	***p*-value**
a.	Allahverdi	135	2	0.890	0.217	0.783	0.113	0.887	0.195	−0.033	0.217	>0.5
	Çakıldak	233	3	0.805	0.302	0.698	0.240	0.760	0.304	0.002	0.062	>0.5
	Karafındık	757	2	0.825	0.221	0.779	0.200	0.800	0.295	0.112	0.005	>0.9
	Mincane	421	2	0.820	0.315	0.685	0.206	0.794	0.316	0.002	0.144	>0.5
	Palaz	81	5	0.791	0.326	0.674	0.268	0.732	0.308	−0.027	0.085	>0.5
	Sarıfındık	255	5	0.779	0.305	0.695	0.290	0.710	0.334	0.061	0.005	>0.9
	Sivri	265	4	0.812	0.303	0.697	0.228	0.772	0.280	−0.031	0.127	>0.5
	Tombul	282	8	0.778	0.307	0.693	0.287	0.714	0.316	0.037	0.017	>0.5
	Yomra	674	2	0.852	0.274	0.726	0.162	0.838	0.276	0.004	0.184	>0.5
b.	Res	662	8	0.805	0.229	0.771	0.270	0.730	0.292	0.161	0.066	>0.5
	Susceptible	625	13	0.813	0.234	0.766	0.253	0.747	0.286	0.114	0.025	>0.9

The summary of population genetics statistics for resistant vs. susceptible accessions showed that the number of private alleles was similar in both, and higher than in varietal sub-populations of similar size (Table 2). Most of the population-wide statistics were similar for both groups, although the F_IS_ value of resistant accessions was higher than for susceptible accessions. Unlike the cultivated sub-populations, the resistant and susceptible groups had lower observed heterozygosity than expected heterozygosity, and correspondingly higher F_IS_. This is consistent with this group comprising largely wild accessions, that are generally more inbred than cultivated varieties (Helmstetter et al., 2020).

### Population Structure

**FIGURE 3:**
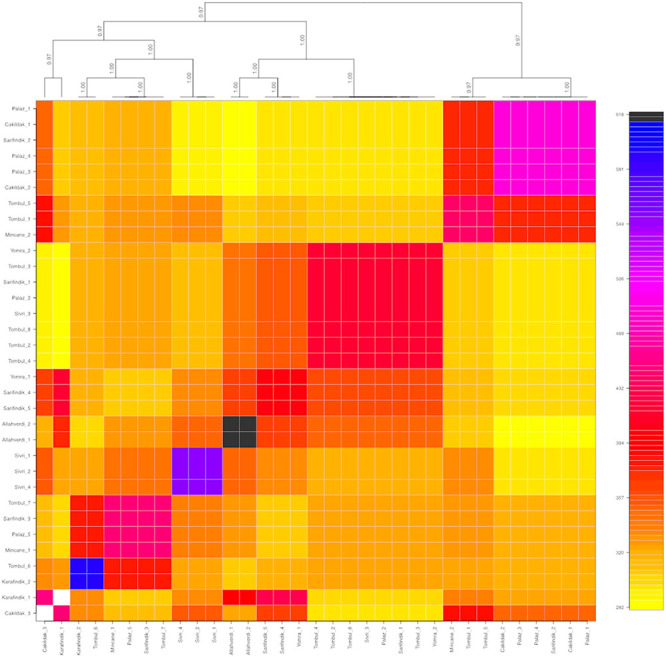
Co-ancestry heatmap and cladogram of hazelnut individuals composing of nine different varieties: ‘Allahverdi’, ‘Çakıldak’, ‘Karafındık’, ‘Mincane’, ‘Palaz’, ‘Sarıfındık’, ‘Sivri’, ‘Tombul’, and ‘Yomra’. The color scale on the right shows the degree of shared co-ancestry. Bootstrap values for the cladogram are indicated on the branches.

The population structure among 32 individuals of named Turkish hazelnut varieties was inferred using *fineRADStructure* software package, which aims to discover conserved haplotypes in order to understand the co-ancestry between individuals. Nearest neighbor calculations using the RAD-seq haplotypes were then calculated to cluster the hazelnut individuals. Eight sub-clusters were identified along with two outliers, Çakıldak-3 and Karafındık-1 (Figure 3). Some contamination of the latter of these two is very likely, considering the unusually high number of private alleles and π value of the ‘Karafındık’ sub-population (Table 2). Some individuals from the same variety clustered together, such as ‘Allahverdi’−1 and −2, and ‘Sivri’−1, −2, and −4; ‘Palaz’−1, −3, and −4 clustered with ‘Çakıldak’−1 and −2. The 8 ‘Tombul’ accessions included 4 that fell into the same broad cluster (‘Tombul’−2, −3, −4, and −8) but the other four were spread among three different clusters. Similarly the ‘Mincane’, ‘Sarıfındık’, and ‘Yomra’ accessions were dispersed among multiple clusters. ‘Sarıfındık’, ‘Yomra’, and ‘Karafındık’ were collected from the western Black Sea region; however they were originally propagated from eastern Black Sea hazelnut varieties through migrating growers (Erdoğan, 2018). Therefore, it is unsurprising that they were not clustered according to their recent geographical origin. This is particularly striking in the case of ‘Sarıfındık’ for which five representatives were dispersed among four genetic clusters, despite all being collected from a small group of orchards in the same local area (Figure 1).

### Phylogenetic Analysis and F_ST_ Values Between Hazelnut Varieties

**FIGURE 4:**
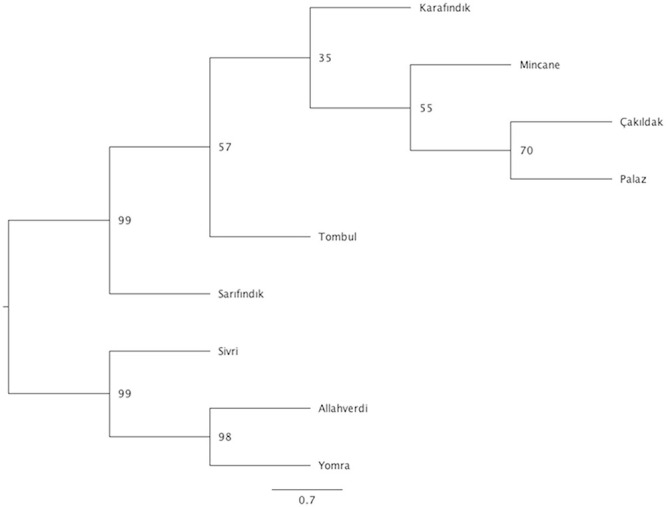
Phylogenetic tree of hazelnut varieties. Bootstrapping values are indicated on the nodes, while scale bar indicates genetic distance. No. of individuals for ‘Allahverdi’, 2; ‘Çakıldak’, 3; ‘Karafındık’, 2; ‘Mincane’, 2; ‘Palaz’, 5; ‘Sarıfındık’, 5; ‘Sivri’, 4; ‘Tombul’, 8; and ‘Yomra’, 2.

Phylogenetic analysis was performed to infer evolutionary relationships between Turkish hazelnut varieties using the SNP loci that were common to every variety with a Maximum Likelihood method. The results showed two different clades: Clade 1 included ‘Sivri’, ‘Allahverdi’, and ‘Yomra’; Clade 2 included ‘Tombul’, ‘Sarıfındık’, ‘Mincane’, ‘Karafındık’, ‘Çakıldak’, and ‘Palaz’, although ‘Sarıfındık’ diverged from the rest of the clade with 99% bootstrap support (Figure 4).

The fixation index (F_ST_) values reflect the degree of genetic differentiation between sub-populations, and similarly indicated closer genetic relationships between ‘Tombul’, ‘Sarıfındık’, ‘Mincane’, ‘Çakıldak’, and ‘Palaz’ (Table 3). ‘Sarıfındık’ and ‘Mincane’ are thought to be closely related as they were both originated from the same variety, propagated under different names in the western and eastern Black Sea regions respectively; however, the measures used here suggested that both are now genetically closer to ‘Tombul’ than each other. Hazelnut propagation in Turkey often uses not a single clone, but a group of clones with similar morphological and physiological characteristics (İslam, 2003). Therefore during the selection and propagation of ‘Mincane’ and ‘Sarıfındık’, ‘Tombul’ suckers might have been included among those individuals. On the other hand, different environmental conditions between the two regions could have driven the genetic differentiation of ‘Sarıfındık’ from the rest of the group. Additionally, hazelnut selection and propagation in Turkey were always conducted around the eastern Black Sea region so it is likely that ‘Tombul’, ‘Mincane’, ‘Çakıldak’, and ‘Palaz’ have been grown together (H. Irfan Balık, personal communication). The divergences between these varieties were not well supported in the phylogenetic analysis (Figure 4), indicating that they share recent common ancestry.

**TABLE 3:** Inter-varietal F_ST_ values matrix.

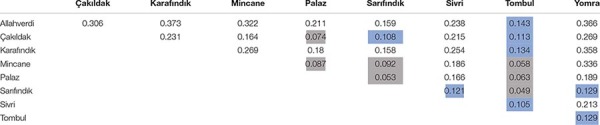

*F_*ST*_ values from 0.1 to 0.15 are indicated in blue, those in gray are F_*ST*_ values below 0.1.*

### Private and Common Alleles and Gene Annotation

An ideal variety-specific marker should be a locus that is present in all hazelnut varieties, but that has alleles that are unique to one or more varieties. Therefore, an investigation was conducted to identify “private” and “common” alleles belonging to each hazelnut varietal sub-population, and private alleles found specifically in resistant accessions. Private alleles were defined as those which differed from the reference (‘Tombul’) genome and were only found in one sub-population; therefore, for the varietal sub-populations these contain variety-specific SNPs, while private alleles in the mildew-resistant sub-population might be linked to genes involved in pathogen defense mechanisms. “Common” alleles were also different from the reference genome, but found in more than one varietal sub-population.

**FIGURE 5:**
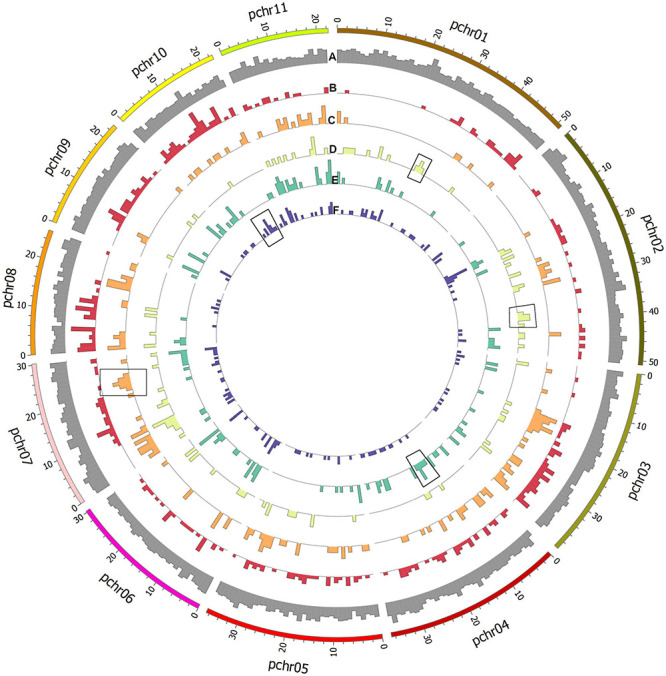
Circular plot showing histograms of the number of assembled Stacks loci mapped to the genome assembly of *C. avellana* var. ‘Tombul’. Outermost ring shows the relative density of all loci **(A)** by genome position. Polymorphic loci were selected, and alleles that differed from the reference genome and were present in specific populations were mapped for each variety. Working from outside to the center of the figure, alternative alleles for the **(B)** ‘Tombul’, **(C)** ‘Sivri’, **(D)** ‘Sarıfındık’, **(E)** ‘Palaz’, and **(F)** ‘Çakıldak’ varieties are shown. These include both alleles that are unique to a certain variety (private alleles), and those which are shared between some varieties but absent in others.

All private and common alleles found in each variety were also mapped to the reference genome (Figure 5). Stacks loci were approximately evenly distributed throughout the genome, but polymorphic sites were distributed differently in each variety; for example, the ‘Tombul’ sub-population had a higher density of polymorphisms on chromosomes 8, 9, and 10, but very few on the short arms of chromosomes 1, 2, and 3 (Figures 5A,B). These observations suggest that genetic diversity may be localized in specific chromosome regions, which could be diagnostic for each variety.

For example, ‘Sivri’ differed most clearly from the other varieties in a block near the distal end of chromosome 7, while ‘Sarıfındık’ contained blocks of variety-specific alleles that mapped to chromosomes 1 and 2. Many of the alternative alleles were shared between ‘Çakıldak’ and ‘Palaz’, reflecting their recent common ancestry (Figures 3, 4), but there were also blocks that could distinguish between them on chromosomes 4 and 10, respectively. These variety-specific blocks could contain genes that confer distinctive phenotypic characteristics, even when other parts of the genome vary throughout breeding.

In order to test whether the polymorphisms found in private alleles reported here could have direct functional effects, their sequences were also mapped to the ‘Tombul’ reference genome (see section ‘Materials and Methods’). Private alleles that fell within predicted gene models were identified, and the Gene Ontology annotations of these genes noted for further evaluation.

### Private Alleles

The private alleles noted in Table 2 were identified to detect variety-specific SNPs. These were further reduced to those that could be most useful as diagnostic SNPs by selecting private alleles that were homozygous for the non-reference allele or heterozygous across all members of a sub-population (described in Supplementary Table 1). In total, 101 different alleles were found with at least 1 from each variety; 57 private alleles were 100% homozygous, showing no diversity within their sub-population, suggesting that they might be fixed in the relevant variety. The other 44 alleles were 100% heterozygous within their variety, which indicates that the alternative SNP allele could be diagnostic for that variety, but is not yet fixed in the population. Comparison of these private allele SNPs with the ‘Tombul’ genome found that 59 of them fell within predicted gene coding sequences and therefore may have a direct functional effect (Supplementary Table 2).

Three private alleles from the variety ‘Allahverdi’ (Locus IDs 6636, 6639, and 7133) were found in Stacks loci that were present in all the hazelnut varieties tested, making these loci ideal as diagnostic markers for ‘Allahverdi’. However, the rest of the loci harboring private alleles were only sequenced in a subset of the population. This might be due to differences in efficiency of the ddRAD-seq library preparation between samples, or point mutations in restriction sites causing some of these loci not to be detected during ddRAD-seq analysis (allele dropout). Some of these may also be useful as variety-specific markers, but would need to be validated on a larger population first.

### Common Alleles

The “common” alleles listed in Table 4 were contained by Stacks loci that were successfully sequenced from all individuals. For this analysis, the ‘Tombul’ group was split into two sub-populations to reflect the observed co-ancestry (Figure 3); ‘Tombul_1’ included individuals Tombul-1, −5, −6, and −7, while ‘Tombul_2’ included Tombul-2, −3, −4, and −8. It was observed that very few alternative alleles were shared between these sub-populations.

**TABLE 4:** List of common alleles showing polymorphisms in specific varieties.

**Locus ID**	**Chr**	**BP**	**Col**	**Var**	**P Nuc**	**Q Nuc**	**N**	**P**	**Obs Het**	**Obs Hom**	**Exp Het**	**Exp Hom**	**π**	**F_IS_**
46	1	10900290	111	Allahverdi	–	T	2	0	0	1	0	1	0	0
			111	Sarıfındık	A	T	5	0.6	0	1	0.48	0.52	0.5333	1
			111	*All others*	A	–	25	1	0	1	0	1	0	0
4404	2	42326313	131	Allahverdi	–	T	2	0	0	1	0	1	0	0
			131	Mincane	C	T	2	0.5	0	1	0.5	0.5	0.6667	1
			131	Tombul_2	C	T	4	0.75	0	1	0.375	0.625	0.4286	1
			131	*All others*	C	–	24	1	0	1	0	1	0	0
6415	3	30379868	126	Allahverdi	C	T	2	0.5	1	0	0.5	0.5	0.6667	−0.5
			126	Karafındık	C	T	2	0.5	0	1	0.5	0.5	0.6667	1
			126	*All others*	C	–	28	1	0	1	0	1	0	0
8388	4	29260698	47	Karafındık	G	A	2	0.5	0	1	0.5	0.5	0.6667	1
			47	Tombul_1	G	A	4	0.75	0	1	0.375	0.625	0.4286	1
			47	*All others*	G	–	26	1	0	1	0	1	0	0
14093	7	7220272	30	Sarıfındık	T	C	5	0.8	0	1	0.32	0.68	0.3556	1
			30	Yomra	T	C	2	0.5	0	1	0.5	0.5	0.6667	1
			30	*All others*	T	–	25	1	0	1	0	1	0	0
15955	9	16105828	118	Karafındık	T	A	2	0.5	0	1	0.5	0.5	0.6667	1
			118	Tombul_1	T	A	4	0.75	0	1	0.375	0.625	0.4286	1
			118	*All others*	T	–	26	1	0	1	0	1	0	0

*Position mapped in genome is given by chromosome (Chr), start position of locus (BP), position of the SNP within locus (Col), variety name (Var), reference nucleotide (P Nuc), alternative nucleotide (Q Nuc), number of individuals that contain the locus, proportion of the reference nucleotide in these individuals (P), observed (Obs) and expected (Exp) heterozygosity (Het) and homozygosity (Hom), nucleotide diversity (π), and inbreeding coefficient (F_*IS*_).*

Six common loci harboring polymorphic alleles were found on chromosomes 1, 2, 3, 4, 7, and 9, in which the majority of varieties had the reference nucleotide SNP position, but some varieties showed an alternative allele (Table 4). Hence these alternative nucleotides could be used as SNP markers to partially identify specific varieties. For example, a “T” allele in the polymorphic site of Locus 46 was observed in both ‘Allahverdi’ and some of the ‘Sarıfındık’ individuals; while a “C” in Locus 14093 was found both in ‘Sarıfındık’ and ‘Yomra’. Therefore, the presence of both of these polymorphisms together could be diagnostic for ‘Sarıfındık’. These SNPs, along with the private alleles noted above, could form the basis of a genetic screening program to confirm the identity of Turkish hazelnut varieties, although they should be validated on larger populations first. Genes associated with these common loci (Table 5) may also be of particular interest, as these loci have been conserved in all the varieties tested during the decades of deliberate selection for hazelnut cultivation in Turkey.

**TABLE 5:** Annotations of genes coinciding with the Common Loci listed in Table 4.

**Locus ID**	**Chr**	**Start**	**End**	**GO terms**	**Annotation descriptions**
46	1	10900178	10901419	GO:0042742; GO:0009611 GO:0002213; GO:0000289 GO:0003676; GO:0008408	Defense response to bacterium; response to wounding; defense response to insect; nuclear-transcribed mRNA poly(A) tail shortening; nucleic acid binding; 3′-5′ exonuclease activity
4404	2	42319235	42320043	GO:0048364; GO:0009506 GO:0030308; GO:0019722 GO:0004871; GO:0005515 GO:0009741; GO:0009505	Root development; plasmodesma negative regulation of cell growth; calcium-mediated signaling signal transducer activity; protein binding response to brassinosteroid; plant-type cell wall
6415	3	30379371	30382243	No GO annotation	Closest match (BLASTn, 67.8% identity): Probable serine/threonine-protein kinase DDB_G0282963 [Pyrus × bretschneideri]
8388	4	29259074	29261788	No GO annotation	Closest match (BLASTn, 57.5% identity): Pentatricopeptide repeat-containing protein At4g14190, chloroplastic [*Prunus mume*]
14093	7	7219073	7222709	GO:0005509; GO:0005634 GO:0009409; GO:0006355 GO:0003677	Calcium ion binding; nucleus response to cold; regulation of transcription, DNA-templated DNA binding
15955	9	16105261	16107681	GO:0003824; GO:0044763 GO:0071704; GO:0044710 GO:0044237	Catalytic activity; single-organism cellular process; organic substance metabolic process; single-organism metabolic process cellular metabolic process

### Private Alleles of Resistant and Susceptible Accessions

Private alleles were identified between eight powdery-mildew resistant and thirteen susceptible accessions. None of these alleles were in all members of either group; this is expected owing to the greater genetic diversity between wild accessions compared with cultivated varieties. However, five private alleles were selected for which the alternative allele frequency was >50% in one group, but absent in the other (Table 6). Locus IDs 781, 22018, 9218, and 18249 contained private alleles from the resistant group. Locus 781 contained a C:T polymorphism that was present in four resistant accessions, three of which were homozygous. Locus 22018 was homozygous in five resistant accessions for a T:A polymorphism. Most frequently, 7/8 resistant accessions showed a C:T polymorphism in locus 9218; all but one of these were homozygous, making this the most promising candidate disease resistance locus. Furthermore, it overlaps with a gene that is predicted to be involved in stress response signaling (Table 7), although this may be coincidental; further research is needed to determine whether specific alleles of this gene, or others near this locus, could contribute to disease resistance. Locus 18249 was also found in four resistant accessions, exhibiting a T:C polymorphism with 50% homozygosity. The repeated occurrence of these polymorphisms in resistant accessions offers a possibility that they are linked to genes involved in powdery mildew resistance; in contrast, the single private allele found in the majority of susceptible accessions, in Locus 21961, could possibly be associated with powdery mildew susceptibility. Comparing with gene models from the reference genome found that most of these polymorphisms fell in inter-genic regions; however, the closest predicted gene models to each private allele were identified and are given in Table 7.

**TABLE 6:** List of private alleles that were specifically polymorphic in either Resistant or Susceptible group.

**Locus ID**	**Chr**	**BP**	**Col**	**Group**	**P Nuc**	**Q Nuc**	**N**	**P**	**Obs Het**	**Obs Hom**	**Exp Het**	**Exp Hom**	**π**	**F_IS_**
781	1	22594282	18	Resistant	C	T	4	0.125	0.250	0.750	0.219	0.781	0.250	0.000
22018	3	3188310	139	Resistant	T	A	5	0.400	0.000	1.000	0.480	0.520	0.533	1.000
9218	5	10896200	127	Resistant	C	T	7	0.357	0.143	0.857	0.459	0.541	0.495	0.711
18249	11	17261698	72	Resistant	T	C	4	0.250	0.500	0.500	0.375	0.625	0.429	−0.167
21961	3	30158075	40	Susceptible	C	T	9	0.333	0.000	1.000	0.444	0.556	0.471	1.000

*Position mapped in genome is given by chromosome (Chr), start position of locus (BP), position of the SNP within locus (Col), group, reference nucleotide (P Nuc), alternative nucleotide (Q Nuc), number of individuals that contain the locus, proportion of the reference nucleotide in these individuals (P), observed (Obs) and expected (Exp) heterozygosity (Het) and homozygosity (Hom), nucleotide diversity (π), and inbreeding coefficient (F_*IS*_).*

**TABLE 7:** Annotations of genes coinciding with the private alleles listed in Table 6.

**Locus ID**	**Chr**	**Start**	**End**	**GO terms**	**Annotation descriptions**
22018	3	3182569	3186923	GO:0046983; GO:0048446; GO:0043565; GO:0006355	Protein dimerization activity; petal morphogenesis; sequence-specific DNA binding; regulation of transcription, DNA-templated
9218	5	10894238	10894792	GO:0042538; GO:0006572; GO:0001560; GO:0009753; GO:0048046; GO:0005829; GO:0009611; GO:0005773; GO:0004838; GO:0009737; GO:0016020	Hyperosmotic salinity response; tyrosine catabolic process; regulation of cell growth by extracellular stimulus; response to jasmonic acid; apoplast; cytosol; response to wounding; vacuole; L-tyrosine:2-oxoglutarate aminotransferase activity; response to abscisic acid; membrane
18249	11	17251842	17258608	GO:0009611; GO:0009753; GO:0005215; GO:0006857; GO:0016020	Response to wounding; response to jasmonic acid; transporter activity; oligopeptide transport; membrane
21961	3	30160976	30167736	No GO annotation	Closest match (BLASTn, 73.9% identity): uncharacterized protein LOC100246151 isoform X1 [*Vitis vinifera*]

## Discussion

Marker screening has previously been performed for Turkish hazelnut varieties, using microsatellite markers were to investigate their genetic diversity (Kafkas et al., 2009; Erdoğan et al., 2010; Gürcan et al., 2010; Öztürk et al., 2017a, b). SNP markers were used in this research to provide a higher resolution for DNA fingerprinting of diverse varieties, and to understand the population structure of cultivated hazelnut trees in Turkey. Representatives of nine commercial hazelnut varieties collected from multiple locations both from the Giresun Hazelnut Research Institute collection and private orchards were sequenced, and their SNP profiles analyzed using population genetics methods. In total 1,048,575 SNPs were discovered across all individuals, greatly increasing the number of known nucleotide polymorphisms in hazelnut. Previously, Torello Marinoni et al. (2018) identified 9,999 SNPs using a Genotyping-by-Sequencing approach, and generated saturated linkage maps for a segregating population of two parents, Tonda Gentile delle Langhe and Merveille de Bollwiller. The SNPs reported here are also potentially valuable for genetic and QTL mapping, although only a minority of loci (10,645) were consistently retrieved from all individuals. This indicates a high level of genetic diversity between individuals, and high levels of heterozygosity found in cultivated accessions (Table 2) also necessitate careful selection of potential molecular markers.

Our previous study found that cultivated Turkish hazelnuts could be grouped into three broad genetic clusters, but that these clusters did not correspond to cultivated variety names; it was also observed that there was no evidence for a strong domestication bottleneck in hazelnut, but that domestication is a gradual process that is still ongoing (Helmstetter et al., 2020). The data presented here explores the implications of this genetic history on individuals at the orchard level, which can help define strategies for breeding and genetic improvement.

### Heterozygosity Is Higher in Hazelnut Cultivars Than Resistant and Susceptible Hazelnut Accessions

Domestication differs between annual and perennial plants. While sexual reproduction is the usual propagation strategy for annual plants, vegetative propagation is often a common practice for perennial plants (Miller and Gross, 2011). The reasons for preferring clonal propagation are the long juvenile stage, and self-incompatibility (Migicovsky et al., 2021). It is therefore favorable for breeders to clonally propagate plants with desirable traits in order to maintain them. Hazelnut cultivation in Turkey is performed largely through vegetative propagation. Growers usually exchange and migrate suckers of hazelnut varieties based on their pomological and morphological appearance across the Black Sea region, which has led to propagation of trees with similar phenotypes, health and nut quality for centuries (Erdoğan et al., 2010).

The genome of cultivated Turkish hazelnuts consisted of approximately 21–32% heterozygous and 67–78% homozygous alleles, depending on the variety; whereas that of resistant and susceptible wild accessions consisted of 22–23% heterozygous and 76–77% homozygous alleles (Table 2). The observed heterozygosity among Turkish cultivated varieties could result from vegetative propagation of heterozygous individuals that were initially produced by outcrossing. These plants might have improved phenotypes through heterosis, so that growers favor heterozygous varieties in the course of selective propagation practices.

On the other hand, heterozygosity was not as high in the mildew-resistant and susceptible accessions, which were largely taken from un-cultivated trees. Lower observed than expected heterozygosity in these accessions suggests that wild hazels may show inbreeding over time; the population-wide genetic diversity means that self-incompatibility is less frequent than within a domesticated variety, although many Turkish varieties do still produce some nuts on selfing, showing that self-incompatibility is not complete (Balık and Beyhan, 2019). This finding is supported by the population genetics study conducted on wild hazelnut accessions collected from Ireland (Brown et al., 2016). Uncultivated hazelnut trees might naturally mate within a limited area leading to inbreeding, which could limit gene flow and increase homozygosity. On the other hand, biparental inbreeding may increase genetic drift (Duminil et al., 2009), giving a greater probability for novel mutations such as those conferring powdery mildew resistance to develop.

### Use of Private and Common Alleles as Molecular Markers

Marker identification for desirable traits will be challenging for phenotypically similar but genetically diverse hazelnut varieties with many heterozygous loci, since there may be multiple alleles within a single variety that confer a trait of interest. Determining firm trait-marker associations is beyond the scope of this study; however, the SNPs reported here for Turkish hazelnut cultivars and resistant wild accessions provide an important basis for future association mapping studies, and the private alleles also might be useful as diagnostic markers for specific varieties and mildew resistance, respectively. The fact that no single polymorphism was common to all the mildew-resistant individuals shows that this resistance is not conferred by a single dominant resistance (R) gene; however there may be multiple R genes in different individuals. Also, varying levels of partial/quantitative resistance have been observed across the Turkish hazelnut population (Lucas et al., 2018), suggesting that a genome-wide association study could be an effective approach to mapping this trait.

### Hazelnut Propagation Practices in Turkey Contribute to Polymorphisms Arising Within the Varieties

Hazelnut classification in Turkey is primarily based on the shape of nut and quality of kernel (Kafkas et al., 2009; Erdoğan et al., 2010; Balık et al., 2018). A good quality hazelnut has a round shape, a high oil content, a high blanching rate, and a rich and aromatic taste. Therefore, the ‘Tombul’ variety, known for its nut quality, has been selected for these complex phenotypes and vegetatively propagated across the Black Sea region. The ‘Tombul’ individuals sequenced in this study had high nucleotide diversity (Table 2), did not cluster in the co-ancestry matrix (Figure 3) and contained many polymorphisms compared to the reference ‘Tombul’ genome (Figure 5). This revealed that individuals within the ‘Tombul’ population were diversified and admixed, which has already mentioned by previous studies (İslam, 2003; Kafkas et al., 2009; Gürcan et al., 2010; Balık et al., 2018; Helmstetter et al., 2020). These observations suggest that hazelnuts currently propagated as ‘Tombul’ are a complex of different genetic varieties with convergent phenotypes. This is consistent with current ‘Tombul’ orchards having been selected by growers who collected and propagated suckers of representative ‘Tombul’-like individuals, but not from a single clone. Over time these practices might lead to propagation of a mixture of different clones for which the physical appearance seems very much alike (Balık et al., 2018). Therefore, the genetic diversity within the cultivated varieties might originate from these traditional propagation practices.

Another hypothesis is that somatic mutations in meristem tissues might be a reason for genetic diversification in hazelnut cultivars (McKey et al., 2010). This is realized when a cell lineage mutates and out-competes other cell lineages in the same tissue through an advantage in cell proliferation. This is a very common occurrence for other clonally propagated crops such as grape and apple. As a grape variety, Pinot has been extensively cloned from the mother plant, but during the course of vegetative propagation somatic mutations have led to diversify the variety and produced Pinot Blanc, Pinot Gris or Pinot Teinturier (Myles et al., 2011). A similar genetic mutation could be also observed in the most cultivated commercial apple variety sports such as Wijcik McIntosh, a sport of McIntosh, which has been selected for high-density planting (Migicovsky et al., 2021). Therefore, somatic mutations might have happened over the decades of clonal cultivation of domesticated hazelnut, which could cause increased genetic diversity within the varieties (Helmstetter et al., 2020).

The nucleotide diversity (π) in most of the cultivated varieties was similar to the wild accessions (Table 2), suggesting that hazelnut has avoided the domestication bottleneck observed in many annual species (Cornille et al., 2012; Helmstetter et al., 2020). Consequently, these varieties may have preserved enough genetic diversity within the variety to adapt to changing environmental stress conditions. Although a highly productive elite line might provide great benefit for growers, preserving the genetic diversity in the varieties is also vital for long-term sustainability.

On the other hand, the variety ‘Allahverdi’ showed characteristics much more typical of a true cultivar, showing the lowest nucleotide diversity and highest co-ancestry between individuals (Figure 1). ‘Allahverdi’ was released in 2013 as a selection from the genotype collection at the Giresun Hazelnut Research Institute characterized by high, stable yield and late leaf opening. Therefore, it is much closer to a clonal cultivar than the other varieties considered here; as a result, it was also easiest to find private alleles that are unique to ‘Allahverdi’, which could facilitate molecular identification and breed protection of this valuable variety.

Regarding the phylogenetic tree, ‘Allahverdi’, ‘Yomra’, and ‘Sivri’ diverged from ‘Sarıfındık’, ‘Tombul’, ‘Karafındık’, ‘Mincane’, ‘Palaz’, and ‘Çakıldak’ (Figure 4). The difference between ‘Tombul’ and ‘Allahverdi’ illustrates how different propagation approaches affect the development of elite varieties in such plant species. These results indicate that ‘Tombul’ should not be considered as a cultivar due to high genetic diversity within the variety; however, as ‘Tombul’ is one of the most economically important varieties in Turkey, there would be considerable value in initiating an elite cultivar breeding program using selections from this variety as primary parents.

### Pollinators Affect Quality Traits in Clonally Propagated Hazelnut Varieties

The propagation system of a plant influences its genetic population structure. Growers use either seed propagation or vegetative (clonal) propagation in order to produce breeding lines (Zohary, 2004). Seed propagation is sexual reproduction, so plants that are propagated through seeds undergo a series of recombination and selection events throughout their breeding history; therefore, inbreeding is required to ensure trait stability (Zohary, 2004). Clonally propagated plants are usually perennials, outcrossers, and increasingly heterozygous individuals may be selected as a strategy to avoid the effects of deleterious alleles that might have accumulated through the years (McKey et al., 2010; Miller and Gross, 2011). Hazelnut trees fit very well with the definition of clonally propagated fruit trees. They have a very long generation time which is up to 8 years to achieve full maturity, and clonal propagation is the only way rapidly to multiply a hazelnut tree with desired traits (Lucas et al., 2020). Selection against inbreeding depression might have been performed over the years of hazelnut cultivation in Turkey, as a lower inbreeding coefficient was observed in hazelnut varieties than in the wild accessions (Table 2).

The outcrossing nature of hazelnut necessitates the planting of fertile and correct pollinators in the vicinity of the primary production trees, in order to set the nuts. Turkish hazelnut varieties show partial self-incompatibility and could still set seeds when they are selfed, however this greatly affects the nut quality and thus reduces the hazelnut productivity (Balık and Beyhan, 2019). For this reason, cross-pollination with suitable pollinators is very important for a good quality hazelnut.

## Conclusion

The investigation of genetic diversity of Turkish hazelnut varieties showed many of them have high intra-varietal diversity, and that several varieties are genetically admixed. We also identified high genetic diversity within the variety itself. This reflects the lack of a long term breeding program for producing elite lines of the best quality nuts, such as ‘Tombul’. Although protecting genetic diversity is crucial for adaptation to changing environmental conditions, generating elite lines has the potential to increase the commercial value of hazelnut production. We were able to define diagnostic SNPs for most varieties that can provide reliable identification in the field, and facilitate marker-assisted selection in breeding programs.

The comparative genetic analysis of resistant and susceptible accessions provided us promising loci that could be used as powdery mildew resistance associated markers. However, no single polymorphism was found in all resistant (or susceptible) accessions. To explore this possibility further, exploration of natural genetic variation among diverse hazelnut accessions through a genome-wide association mapping approach would allow the discovery of mildew disease resistance traits, along with other genes important improve hazelnut cultivation in Turkey.

## Data Availability Statement

The datasets presented in this study can be found in online repositories. The names of the repository/repositories and accession number(s) can be found below: https://www.ebi.ac.uk/ena, PRJEB32239.

## Author Contributions

NO-E collected samples, carried out experimental work, analyzed data, and drafted the manuscript. AH carried out experimental work, analyzed data, and commented on the manuscript. Aİ assisted with data analysis and preparing the manuscript. RB and SL conceived the study, managed the project, and revised the manuscript. SL also collected samples and developed experimental and computational methods. All authors contributed to the article and approved the submitted version.

## Conflict of Interest

The authors declare that the research was conducted in the absence of any commercial or financial relationships that could be construed as a potential conflict of interest.
