# Supplementary material for: Unraveling Genetic Diversity Amongst European Hazelnut (Corylus avellana L.) Varieties in Turkey
Source: Front Plant Sci. 2021 Jul 1;12:661274. doi: 10.3389/fpls.2021.661274 (PMC8282208; doi:10.3389/fpls.2021.661274)
Supplement: Supplementary file 1 [file Data_Sheet_1.zip › Supplementary Figure 1.PDF]

pstacks

- Aligned data were grouped into loci and polymorphic nucleotide sites were identified for each individual. Default settings were used.

cstacks

- Loci were grouped together across each individual. A consensus catalogue was created. The number of mismatches allowed between sample loci when building the catalogue (n) was set to 3.

sstacks

- Loci of each individual were tested if they matched against the consensus catalogue. Default settings were used.

rxstacks

- Genotype and haplotype corrections were made based on the population-wide accumulated data. The consensus catalog loci were filtered based on the mean log likelihood of the catalogue locus in the population (--lnl_filter). The minimum log likelihood was set to -20 (--lnl_lim). The proportion of loci in population was set to 0.25 that must be confounded relative to the catalog locus (--conf_lim). Non-biological haplotypes were pruned out (--prune_haplo). *cstacks* and *sstacks* were rerun to build the consensus catalogue loci and to test the matches across the individuals and the catalogue.

populations

- The population genetic statistics were calculated based on the population map showing which individuals belong to which population. Individuals that belong to a variety were grouped within the same population. The minimum number of populations a locus must be present in to process a locus was set to 1 (--p). The minimum percentage of individuals in a population required to process a locus for that was set to 50% (--r). The minor allele frequency was set to 0.05 (--min_maf). F statistics (Fixation index (F_{ST})) were calculated, and the maximum cut-off *p*-value was set to 0.05 (--fstats and --p_value_cut-off). Private alleles were identified specifically for every variety. The proportion of heterozygous and homozygous alleles observed in every variety (Obs Het and Obs Hom), and the heterozygosity (2*pq*) and the homozygosity (1-2*pq*) expected under Hardy-Weinberg equilibrium (Exp Het and Exp Hom), the frequency of the most frequent allele at each locus (P), the nucleotide diversity (Pi), and the inbreeding coefficient of individual relation to the subpopulation (F_{IS}) were calculated for all identified alleles in every variety. A phylip file was created to show the variant sites across varieties.
